# Diuron in Water: Functional Toxicity and Intracellular Detoxification Patterns of Active Concentrations Assayed in Tandem by a Yeast-Based Probe

**DOI:** 10.3390/ijerph120403731

**Published:** 2015-04-01

**Authors:** Roberto Dragone, Rachel Cheng, Gerardo Grasso, Chiara Frazzoli

**Affiliations:** 1 Institute for Nanostructured Materials (ISMN), National Research Council, 00185 Rome, Italy; 2 Department of Agriculture, Forestry, Nature and Energy (DAFNE), Tuscia University, 01100 Viterbo, Italy; E-Mail: rachel.cheng@unitus.it; 3 External Relations Office, National Institute of Health, 00162 Rome, Italy; E-Mail: chiara.frazzoli@iss.it; 4 Department of Agricultural, Environmental and Food Sciences, University of Molise, 86100 Campobasso, Italy; E-Mail: gerardo.grasso@hotmail.it

**Keywords:** herbicide, respirometric biosensor, mitochondrial toxicity

## Abstract

A study on the acute and chronic effects of the herbicide diuron was carried out. The test, basing on a yeast cell probe, investigated the interference with cellular catabolism and possible self-detoxification capacity of *Saccharomyces cerevisiae*. Aerobic respiration was taken as the toxicological end-point. Percentage interference (%ρ) with cellular respiration was measured in water by increased dissolved O_2_ concentration (ppm) after exposure to different doses. Interference was calculated through the comparison of respiratory activity of exposed and non-exposed cells. Short-term and long-term (6 and 24 h respectively) exposures were also considered. The test for short-term exposure gave positive %ρ values except that for 10^−6^ M (11.11%, 11.76%, 13.33% and 0% for 10^−10^ M, 10^−8^ M, 10^−7^ M and 10^−6^ M respectively). In the case of long-term exposure the test showed positive %ρ values, but less effect than short-term exposure until 10^−8^ M and much higher at 10^−6^ M (7.41%, 8.82%, 11.76% and 6.06% for 10^−10^ M, 10^−8^ M, 10^−7^ M and 10^−6^ M respectively). The findings of aerobic respiration as toxicological end-point were in agreement with known mechanisms of toxicity and intracellular detoxification for both the doses and exposure times employed.

## 1. Introduction

Pesticides are widely used worldwide in agriculture to increase crop yields by killing pests or eliminating diseases. The worldwide consumption of pesticides is about two million tonnes per year (45% of which is used in Europe alone) [1,2]. Clear evidence demonstrates that many of them have toxic effects both on microorganisms [3] and humans [4,5]. According to their use, pesticides are divided into four categories: herbicides, fungicides, insecticides and bactericides. The first review done in Italy on the occurrence of 161 emerging organic compounds (EOCs) in surface waters and groundwaters [1] reported that pesticides and industrial emerging organic compounds were detected in concentrations up to 4.78 × 10^5^ and 15 × 10^6^ ng/L, respectively. According to the review, the phenylurea herbicide diuron (3-(3,4-dichlorophenyl)-1,1-dimethylurea or DCMU), is one of the most frequently detected both in surface and groundwater. This fact is not surprising since diuron is among the most used pesticides in Italy [6]. In Italy, diuron can be used in amounts no higher than 0.5 kg/ha after authorisation by the Italian Health Ministry [7]. Diuron belongs to the urea compounds group and is effective against both mono- and dicotyledonous weeds as well as mosses (mainly absorbed by the roots and translocated following the apoplastic pathway) [8]. The primary mode of action is photosynthesis inhibition by blocking the thylakoid electron transfer chain, but ATP synthesis inhibition is also involved [3]. Inhibition of respiration in the yeast *Saccharomyces cerevisiae* (*S. cerevisiae*) was also reported [9,10,11,12]: in particular, diuron blocks the transport of electrons between cytochromes b and c_1_ (Complex III) of the mitochondrial respiratory chain, inducing the “extra-reduction” of cytochrome b_566_ [10]. 

By selecting *S. cerevisiae* as a respirometric biosensor model (yeast-based probe) for toxicological evaluation [13], in this study we investigated the effect of different *S. cerevisiae* exposure times to different concentrations of diuron in aqueous solution. A yeast-based probe involves three components: (i) a biomediator, *i.e.*, yeast cells, (ii) a transducer, e.g., in our case an amperometric oxygen sensor (oximeter) to measure (instantly and without further procedures) changes in dissolved oxygen (O_2_) concentration correlated with cellular aerobic catabolism, and iii) a continuous data recorder.

Yeast-based probes, like bioassays, have been studied by many groups [3,10,11,12,14]. Differently from yeast-based probes, bioassays use receptors (molecules with affinity for the target) for the identification or quantification of target molecules in a sample; technically, the receptor is mixed with the sample of interest, followed by detection of the receptor-target complex. This step requires the separation between free and bound molecules, and generally the addition of further reagents [15,16]. Yeast-based probes are definitely much quicker than bioassays as there is no need of further steps, *i.e.*, separation of the free and bounded molecules of target analyte. Furthermore, the cellular aerobic respiration in yeast is found to be very sensitive to the presence of various toxic chemicals. Monitoring the change of dissolved O_2_ concentration (linked to mitochondrial activities) allows the rapid assessment of the presence of toxic substances in water [17,18,19,20,21,22,23,24]. 

This study followed the aerobic respiration of *S. cerevisiae* yeast as a toxicological end-point of the acute and chronic effects of diuron. By measuring the change of dissolved O_2_ (in parts per million, ppm) in the presence of known concentrations of diuron, the test observed the interference with respiration and investigated the possible self-detoxification capacity of yeast cells after different exposure times.

## 2. Experimental Section 

The experiments were done based on the works already presented in Frazzoli *et al.* [24]. Diuron inhibits the transport of electrons between cytochromes b and c_1_ of the respiratory chain of *S. cerevisiae* [9,10,11,12]. However, the minimum dose causing this effect has not yet been investigated in the literature, except for a study on the response of *S. cerevisiae* genetically modified to express firefly luciferase to generate a bioluminescent yeast strain to much higher concentrations of diuron (from around 1 to around 40 mg/L) [25]. Therefore, tests were carried out using *S. cerevisiae* exposed to different doses of diuron. According to Directive 89/778/EEC [26], the maximum concentration for each single chemical (including pesticides) in potable water is 0.1 µg/L; using this information, four different concentrations were investigated, *i.e.*, 241.20 µg/L (10^−6^ M, much over the limit), 24.12 µg/L (10^−7^ M, 200 times over the limit), 2.41 µg/L (10^−8^ M, 20 times over the limit) and finally 0.024 µg/L (10^−10^ M, four times below limit). Yeast suspensions were individually exposed to these concentrations for a time period of 6 h. Experiments were done to see the relationship between dose-effect. To study of detoxification ability of the cells, a longer exposure time of 24 h was tested.

### 2.1. Materials, Apparatus, Samples

Dried *S. cerevisiae* cells (*Saccharomyces cerevisiae* yeast, Type II) used in the experiments and herbicide diuron (3-(3,4-dichlorophenyl)-1,1-dimethylurea or DCMU, CAS number 330-54-1) were obtained from Sigma-Aldrich S.r.l. (Milano, Italy). Methanol (assay (GLC) ≥ 99.9%) from Carlo Erba (Cornaredo, Italy) was used to dissolve the herbicide; sodium sulfite used to calibrate the oximeter and D-(+)-glucose monohydrate for microbiology ≥99.0 % used for the test solutions were obtained from Fluka Analytical (Milano, Italy). Sodium azide used to terminate the test was from AMS Biotechnology Ltd. (Bioggio-Lugano, Switzerland). High-purity deionised water (Milli-Q system, Merck Millipore, Billerica, MA, USA) was used for all the standard solutions made in this study. 

A Clark-type oximeter (model 360) from Amel Instruments S.r.l. (Milano, Italy) was used in parallel coupled with an analogue recorder specially made for the experiments carried out in this study (by Amel Instruments S.r.l.) to simultaneously measure and register the dissolved O_2_ concentration in the test solutions in ppm. Data were then analysed with the personal computer, which was connected to the Amel analogue recorder. 

### 2.2. Methods

Using similar method as mentioned in the functional toxicity test of Frazzoli *et al.* [24] but omitting internal blanks, the experiments were prepared and conducted as follows:

- 50 mg ± 1.0 mg dried *S. cerevisiae* cells were rehydrated in test tubes always 12 h prior to the experiment with 10 mL of Milli-Q water without any agitation; no nutrient was added to prevent proliferation of cells.
- 0.5 M of glucose solution with Milli-Q water was prepared at weekly basis for replications of experiments eliminating extra variations of test.
- diuron stock solution, 10^−2^ M, was prepared by adding 60.3 mg of herbicide in 25 mL of methanol: dilutions with water Milli-Q were carried out to obtain 10^−4^ M, 10^−5^ M, 10^−6^ M and 10^−8^ M of diuron respectively.
- two-point calibration of the Clark-type oximeter was completed using: (a) atmospheric oxygen and (b) a sodium sulfite solution (10 g/L). Calibration with atmospheric oxygen was done before every single test because atmospheric oxygen measurements depend on both temperature and barometric pressure [24]. The calibration point with sodium sulfite gives the electrode a zero reference point since this compound binds with the dissolved O_2_.

**Figure 1:**
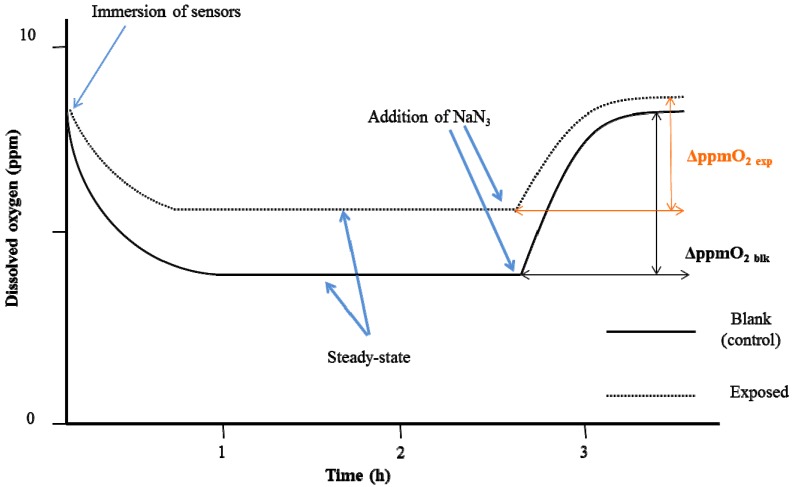
The experimental respirometric curve shows the dissolved oxygen (ppm O_2_) as a function of time in the case of interference with the respiration of the yeast cells.

Measurements were done in an open system with small beakers (25 mL), for both blank (control) and exposed samples, immersed in a water bath to maintain a constant temperature (25.0 ± 0.1 °C) under constant magnet stirring (200 rpm). Total sample volume was 15 mL, which included the glucose solution and 150 µL of yeast suspension that were added in each beaker, followed by 150 µL aliquots of methanolic solutions without diuron and aliquots of methanolic solutions with diuron. Exposure time started once the aliquots of diuron methanolic solution of known concentration (one of the four mentioned above) were added to exposed samples and the equal aliquots of methanolic solutions without diuron were added to control samples. Final concentrations of diuron obtained, by e.g., adding 150 µL of 10^−4^ M diuron solution to 14.7 mL of glucose solution and 150 µL of yeast suspension to obtain an ultimate concentration of 10^−6^ M and for final concentration of 10^−10^ M, 150 µL of 10^−8^ M diuron aliquots was added in each exposed beaker, *etc*. Each test was done with this dilution method to obtain the eventual concentrations in question. Two hours before the end of the exposure, a Clark-type oximeter was immersed into each beaker containing the solutions to stabilise the signals (the first plateau). At the end of the each test (both for 6 and 24 h of exposure time), 100 µL of 0.2 M sodium azide (NaN_3_) was added to fully inhibit the yeast cell respiration [19]. At this point, the amount of dissolved O_2_ rises up to a second plateau (stability of signals being indicated by a fluctuation of less than 0.02 ppm) that corresponds to the amount of dissolved O_2_ in the absence of the yeast cells’ respiration. Respirometric curves for blank (control) and exposed samples of the experiment are shown in Figure 1. 

The analytical parameter used was the variation of the dissolved O_2_ (ΔppmO_2_) between the two plateaux of the experimental curves before and after the addition of NaN_3_ (Figure 1). Means of readings of the dissolved O_2_ concentration at 10 minutes before NaN_3_ (first plateau) addition and once reached the second plateau were calculated, and ΔppmO_2_ was calculated for each experiment. The percentage interference of cellular respiration (%ρ) was calculated with the following algorithm:

%ρ = (1 − (ΔppmO_2 exp_/ΔppmO_2 blk_)) × 100 (1)

where ΔppmO_2 exp_ = mean of variations of the dissolved O_2_ (in ppm) for exposed samples, and ΔppmO_2 blk_ = mean of variations of the dissolved O_2_ (in ppm) for blank samples. All experiments were repeated at least four times (each control and exposed samples had four replicates for each experiment). Relative standard deviations (RSD%) ≤ 20% were calculated for blanks and exposed samples for each experiment for repeatability.

## 3. Results and Discussion

**Figure 2:**
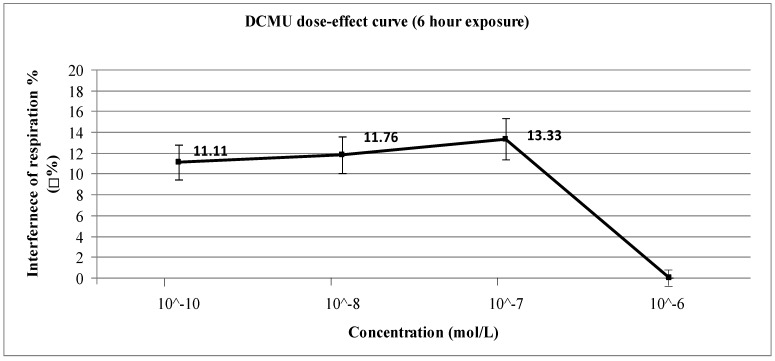
Dose-effect curve for short-term (6 h) exposure to diuron (DCMU).

**Figure 3:**
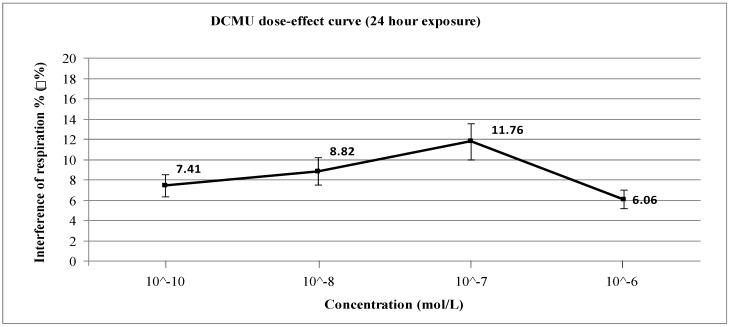
Dose-effect curve for long-term (24 h) exposure to diuron (DCMU).

Even though diuron is reported as a weak inhibitor of cytochrome bc_1_ (Complex III) of the electron-transport chain [27], almost all concentrations investigated caused evident inhibition of cellular respiration after both short-term (Figure 2) and long-term exposure (Figure 3). Statistical tests were done using ANOVA for Randomised Block Design and a significant relationship between exposed and non-exposed samples was found in all concentrations for both exposure periods (*p* < 0.03). The only exception was the 6-h exposure experiments using 10^−6^ M of DCMU because under these conditions there is no inhibition (ρ% = 0): in this case, the statistical test cannot give a significant value because of the absence of any difference between blanks and exposed samples.

Interpretation of results was based on a study of scientific literature. In particular, the results obtained lead us to hypothesise the involvement of at least three intracellular mechanisms already proved for *S. cerevisiae* after exposure to xenobiotics. Namely, these mechanisms are: (i) toxicity mechanism [9,10,11,12,27,28], (ii) cellular defense/detoxification mechanism [29,30], and (iii) cellular repair mechanism [31,32]. 

With regards to the toxicity mechanism, the inhibition observed in our experiments can be explained by the known occurrence of oxidative stress due to reactive oxygen species (ROS). In the presence of some substances as antimycin A, Complex III is one of the major sites for ROS production [33] and diuron is an antimycin-like inhibitor of mitochondrial Complex III in *S. cerevisiae* [9,10,11,12,27,28].

Regarding to the cellular detoxification mechanism, this could be linked to the involvement of a known system present in *S. cerevisiae*, based on the extrusion of xenobiotics (including herbicides) via plasma membrane efflux pumps (called ATP-binding cassette or ABC transporters) [29]; the role of the ABC transporters in diuron resistance has been demonstrated [30].

With regards to the cellular repair mechanism, the induction of an adaptive oxidative stress response due to ROS accumulation could be hypothesised. Yeast cells, as all aerobic organisms, have defense and protective mechanisms against oxidative stress, like ROS scavengers, and damaged molecules repair/removal systems [31,32].

It is noteworthy to mention that each mechanism is characterised by its own kinetics of triggering and action, which are expected to be dose- and exposure time-dependent. These kinetics are critical in the interpretation of the results: according to our interpretation, %ρ values originate from the kinetic combination of the rate of all three mechanisms, like a snapshot at 6 h and 24 h of exposure, respectively. 

In our experiments, detoxification and repair mechanisms seem to be active also under short term exposure (as demonstrated in *S. cerevisiae* cells exposed to the herbicide 2,4-dichlorophenoxyacetic acid) [34]: at an exposure dose of 10^−10^‒10^−7^ M, in fact, the % ρ values decrease when the exposure time increases from 6 to 24 h and at 10^−6^ M, instead, the detoxification/repair mechanisms are already triggered at an exposure time of 6 h, but increases exposure time prevails the toxicity mechanism. 

Finally, in support of the given interpretation of results, the possible occurrence of diuron accumulation at the level of mitochondrial membranes via herbicide interaction with phosphatidylcholine (PC), which is a major phospholipid constituent of mitochondrial membranes in *S. cerevisiae* should also be mentioned [35]. This phenomenon could be linked to the specific interaction between the headgroups of PC molecules and the functional groups of the herbicide molecules [36]. 

Therefore, in the scenarios of 6 h and 24 h exposure our results can be summarised as follows: for *short-term (6 h) exposure*, the results obtained show interference with cellular respiration due to 10^−10^–10^−7^ M diuron; The toxic effect of 10^−6^ M diuron drastically decreases to zero (Figure 2). According to the “three mechanisms scheme”, at 6 h exposure the highest tested concentration (10^−6^ M) is able to trigger the cellular detoxification/repair mechanisms (thus explaining the result %ρ = 0), while at lower doses (10^−10^–10^−7^ M) such triggering is delayed due to a tolerance effect, thus possibly allowing diuron to accumulate in the mitochondria and to exert toxic effect (thus explaining the %ρ values between 11% and 13%). For *long-term (24 h) exposure*, the exposure to 10^−6^ M diuron showed instead a significantly different %ρ value compared to the results obtained under 6 h (Figure 3). This interesting value may be discussed in terms of saturation of cellular detoxification/repair mechanisms. Therefore, under these conditions (10^−6^ M concentration) diuron can accumulate in the mitochondria and exert toxic effects (%ρ = 6.06%), whereas at lower doses the toxic effect is decreased by the speed of detoxification/repair mechanisms (%ρ = 7.41% for 10^−10^ M; %ρ = 8.82% for 10^−8^ M; %ρ = 11.76% for 10^−7^ M).

## 4. Conclusions

The proposed yeast-based probe for diuron is proved to be very sensitive and able to detect it in potable water [26]. The results obtained on the interference on cells’ respiration well match with known mechanisms toxicity, cellular detoxification and repair described in scientific literature for *S. cerevisiae* at the doses and exposure times employed. 
